# Arts Engagement Programs improves Health in Community-Dwelling Older Adults

**DOI:** 10.1093/geroni/igaa057.3086

**Published:** 2020-12-16

**Authors:** Jatin Ambegaonkar, Niyati Dhokai

**Affiliations:** George Mason University, Manassas, Virginia, United States

## Abstract

We examined how different arts engagement programs compared to control affect health in community-dwelling older adults. 64 adults(71.3 + 4.6years; Dance n=23, Music n=17, Control, n=24) took part in free Dance(Ballroom), Music(Ukulele), or Control(Active social conversation) sessions 2 times/week for 10 weeks. We assessed cognition(Montreal-Cognitive-Assessment-MoCA), physical(Short-Physical-Performance-Battery-SPPB), and Health‐Related Quality-of-Life(HRQoL‐SF‐20) 3 times: (1) before(pre), (2) at the end of 10 weeks(post-1) and (3) 1-month after intervention(post-2). Separate 3(Time)x3(Group) ANOVAs and Bonferroni-pairwise-comparisons examined changes across groups and time(p<.05). Participants’ physical health improved equally across groups(p=.4) and over time(p<.001), specifically from pre(10.5 + 1.4) to post-1(10.7 + 1.3; p=.002), and pre to-post-2(11.3 + 1.0;p<.001). Participants’ cognition improved equally across groups(p=.6) and over time(p<.001) from pre(26.3 + 2.8) to post-1(27.3 + 2.5; p=.002), and pre-to-post-2(27.5 + 2.5;p=.001). Participants’ HRQoL remained similar over time(p=.6) and across groups(p=.7). Overall, participants’ health improved after taking part in arts engagement and social conversation programs. Study findings offer insights about successful implementation of arts-engaged programs in community-dwelling older adults.

